# Pasture area increases livestock rabies risk in Rio de Janeiro, Brazil: a geospatial analysis of neurological syndrome cases (2020–2024)

**DOI:** 10.1007/s11250-026-05359-1

**Published:** 2026-10-14

**Authors:** Rafael Veríssimo Monteiro, Renata Falcão Rabello da Costa, Leda Maria Silva Kimura, Glenda Ribeiro de Oliveira, Natiane Rodrigues Viana, Alessandro de Sá Guimarães, Márcio Roberto Silva, Guilherme Nunes de Souza

**Affiliations:** 1 Veterinary Medicine Department, Universidade Federal de Juiz de Fora, Av. Eugênio do Nascimento s/nº - Dom Bosco, Juiz de Fora, MG 36038-330 Brazil; 2 Secretaria de Estado de Agricultura, Pecuária e Abastecimento do Rio de Janeiro. Defesa Agropecuária. Coordenadoria de Defesa Sanitária Animal, Alameda São Boaventura, 770, Fonseca, Niteroi, RJ 20071-004 Brazil; 3 Empresa de Pesquisa Agropecuária do Estado do Rio de Janeiro (PESAGRO- RIO), Alameda São Boaventura, 770 – Fonseca, Niterói, RJ 24120-191 Brazil; 4 Programa de Pós-Graduação em Medicina Veterinária (Clínica e Reprodução Animal) da Universidade Federal Fluminense, Rua Vital Brazil Filho, n. 64, Vital Brasil, Niterói, Rio de Janeiro, 24230-340 Brasil; 5 Empresa Brasileira de Pesquisa Agropecuária – Embrapa Gado de Leite, Av. Eugênio do Nascimento, 610 – Aeroporto, Juiz de Fora, MG 36038-330 Brazil

**Keywords:** *Desmodus rotundus*, Geospatial epidemiology, Lyssavirus, Rabies epidemiology

## Abstract

Animal rabies poses a risk to livestock in Brazil due to transmission by hematophagous bats. The Brazilian sanitary surveillance system mandates rabies testing for any livestock exhibiting signs of neurological syndrome. This geospatial, retrospective, longitudinal, observational study analyzed rabies test results for 204 cattle and 56 horses presenting with neurological syndrome in Rio de Janeiro State, Brazil, from 2020 to 2024. We calculated correlations and linear models between rabies results and municipal land-use types, as well the population of herbivores. Most neurological syndrome cases and positive rabies results occurred in cattle. The final linear model for the number of neurological syndrome cases and positive rabies results incorporated pasture area, shrub area and cattle population. Pasture area was positively correlated with higher numbers (*p* = 0.011 and *p* = 0.005, respectively). Larger shrub area was associated with fewer neurological syndrome reports and fewer positive results (*p* = 0.009 and *p* = 0.010, respectively). Greater cattle population showed a negative association with neurological syndrome notifications and positive detections (*p* = 0.085 and *p* = 0.046, respectively).A higher proportion of positive rabies tests was associated with larger pasture areas (*p* = 0.019) and with lower equine populations (*p* = 0.115). These findings indicate that the lower animal diversity in pasture areas provides few alternative feeding options for *Desmodus rotundus* besides livestock, leading to more neurological syndrome cases and animal rabies infections. A larger shrub area offer feed alternatives other than cattle to the bats and a larger cattle population dilutes the frequency of bat meals across individuals, reducing both neurological syndrome notifications and positive rabies detections.

## Introduction

Rabies is a zoonotic viral disease that causes acute and severe encephalitis in humans and other mammals. The epidemiological cycle of rabies is often divided into four components: aerial (represented by the three species of hematophagous bats), urban, rural, and wildlife (Rocha et al. 2017). Molecular analyses of rabies virus strains circulating in Brazil show that their differentiation is associated with different hosts, such as hematophagous bats, carnivores, and wildlife herbivores. Rabies transmitted by hematophagous *Desmodus rotundus* bats connects rural and wildlife cycles, as they conveniently forage on both cattle and horses, as well on a wide diversity of wild mammals (Brasil. Ministério da Agricultura, Pecuária e Abastecimento, 2009). Such diversity favors the hematophagous bats’ contact with different variants of the rabies virus, in addition to the strain associated with *D. rotundus* itself, which may infect cattle and equine livestock (Brown and Escobar 2023).

It had been documented that the lower the diversity of local vegetation, the lower the vertebrate species diversity (Kremer et al. 2025). That is the case of the cattle livestock environment, the pasture (Filazzola et al. 2020; Perfecto et al. 2023). In this environment the main host for the blood meal of hematophagous bats are cattle, and to a lesser extent, horses; both species that *D. rotundus* will attack. Either species is susceptible to the rabies virus. However, its susceptibility may vary according to the viral strain, host species, inoculation site, age, and immune state of the attacked host (Cunha et al. 2010; White and Razgour 2020). Municipalities with a greater pasture coverage are expected to present a higher number of positive rabies test results, as there is a wider availability of susceptible cattle and horses, and a smaller diversity of other mammal species. The Brazilian Program for Rabies Control in Herbivores stipulates that every bovine and horse presenting clinical signs compatible with encephalitis (Neurological Syndrome - NS) must be tested for rabies, since such a syndrome might have different causes. In Rio de Janeiro, the biological samples collected from these livestock for rabies diagnosis are sent to the State Center of Research in Animal Health ‘Geraldo Manhães Carneiro’, which is a laboratory partnered with the Agricultural Research Company of the State of Rio de Janeiro. Thus, these laboratory results represent all NS cases in domestic herbivores detected by the state government for each municipality and year of sampling, regardless of rabies status (Brasil. Ministério da Agricultura, Pecuária e Abastecimento, 2009). After 21 years with no human cases of rabies in Rio de Janeiro State, Brazil, a case of death was reported due to bat contact in 2006 (Meneguete 2012). Bats, whether hematophagous or not, were identified as the rabies virus host in the state in more than one study (Silva et al. 2007; Mota et al. 2010; Vieira et al. 2010; Cocchi et al. 2024). The disease in bats was considered as emerging in the city of Rio de Janeiro by one scientific publication (Souza et al. 2014). The bats’ constant presence generates a variable number of animal rabies cases per year, but most diagnoses are made in domestic cattle and horses, even though samples from other species are eventually sent to the laboratory and are found positive (Moutinho et al. 2020). The State registered 63% of its municipalities with a minimum of one rabies case in herbivores between 2007 and 2011 (Martins et al. 2013).

This study aims to evaluate the geospatial distribution of bovine and equine rabies tests by municipalities throughout the State of Rio de Janeiro between 2020 and 2024, focusing on answer: (a) whether the tests and their results may have different occurrence frequencies among cattle and horses, municipalities, and years; (b) whether the type of land use in each municipality may correlate or be cause to effect with either the number or the results of the animal rabies tests undertaken in the covered period.

## Material and methods

### Neurological syndrome cases in cattle and horses

The number of animal rabies tests requested for cattle and horses was gathered, as well as their results, from all 92 municipalities of the State of Rio de Janeiro. These data originate from the Animal Health Defense Department, linked to the Superintendency of Agricultural Defense of the State Secretariat of Agriculture, Livestock and Supply of Rio de Janeiro, and the authors have full authorization to use them. These tests were performed on biological samples from cattle and horses presenting with NS. Only negative immunofluorescence samples were submitted to the mice inoculation technique (MIT). The final number of positives was the sum of positives from either test (Brasil. Ministério da Agricultura, Pecuária e Abastecimento, 2009). The age distribution of the tested animals (in months) was compared with a normal distribution using the Cramér–von Mises test. To verify if there were statistical differences between the ages of cattle and horses, the Wilcoxon rank-sum test was used (Sokal and Rohlf 2009). The test results were grouped by host species (bovine or equine), year, and requesting municipality. For each municipality and host, a Ratio of Positives (RP) was created, and it is defined as the number of positive tests divided by the total number of tests undertaken within the period. The cattle and horse populations of each municipality in each year were obtained from SIDRA (Sistema IBGE de Recuperação Automática; Brasil, 2025) and were used as covariates in the linear models defined in this study.

### Distribution and spatial correlation of animal rabies with municipal land use

To determine whether land use in each municipality is related to a higher occurrence of Neurological Syndrome or a higher rabies transmission rate, which is expressed by the number of positives and the Ratio of Positives, we correlated these factors with the areas of different municipal land uses according to the classification made by MapBiomas (Souza et al. 2020). in the State of Rio de Janeiro in each year of this study (2020–2024). Land use was classified into eight groups —Forest, Shrubs, Pastures, Agriculture, Forestry, and Mosaic of Use — while categories such as Water and No Vegetation were excluded from all analyses. The area of each land use was calculated by counting the pixels it occupied in each year and municipality. These pixel counts served as inputs for the correlation analyses and linear models. The Rio de Janeiro state averaged pixel area value was obtained by dividing each municipality’s known total area by the total number of pixels classified, then averaging these values across all municipalities. Non-parametric correlations using Spearman’s test were calculated between each land-use area and the number of NS cases, number of positives, and the ratio of Positives for rabies in each municipality in the pooled data, as cattle and horses use the same type of environment. Maps showing the geospatial municipal distribution of NS cases and Pasture coverage area were generated using QGIS 3.40.6 software, utilizing datum SIRGAS 2000 and the IBGE 2017 geographic database.

### Statistical analysis

One linear model was developed using a backward elimination strategy. The initial model included all six land use types plus bovine and equine population counts as covariates. Three response variables were modeled separately: number of NS cases, number of rabies positive samples, and the ratio of positives. Each response used a different error distribution: NS cases — Poisson; positive counts — negative binomial; ratio of positives — normal. Goodness-of-fit for the chosen distributions was assessed with chi-square tests (*p* > 0.05). The final model was crafted after the regressive removal, searching for a association (considering alpha = 0.05) between land-use type, population of bovine or equine species, and test results. All analyses were performed using R Statistical Software (v4.4.2; R Core Team 2021). Generalized linear models were fitted with the lme4 R package (v1.1.37; Bates et al. 2015). Figures were created with aid of the ggstats R package (v0.13.0; Larmarange 2026).

## Results

### Neurological syndrome cases

The number of NS cases examined in cattle and horses in the official laboratory during this study period, across all analyzed municipalities and animal ages, is summarized in Table 1. The number of tests was higher in cattle than in horses and covered over half of the municipalities in the State of Rio de Janeiro. The age distribution of the tested animals was non-normal, as cattle were younger than horses (Wilcoxon rank test, *p* < 0.001). At least one NS case in bovines or equines occurred in 58 municipalities of Rio de Janeiro State, but 20 of these (34.5%) requested only one diagnosis during the period (Table 1).

**Table 1:** Neurological Syndrome cases and number of municipalities tested for bovine or equine rabies, and the age of tested herbivore, in the State of Rio de Janeiro, within the period of 2020–2024. The proportions were calculated in separate for each herbivore

Herbivore tested	NS cases	Tested municipalities	Population´s age (months)
	(*n* /%)	(*n* / %)	median (IQR)
Bovine	204 / 78.5	44 / 47.8	20 (10–36)
Equine	56 / 21.3	30 / 32.6	39 (19–72)
Total	260	58 / 63.0	24 (11–48)

Obs: The ratios from the “Tested municipalities” column were calculated over a denominator of 92 municipalities in the State of Rio de Janeiro. NS: Neurological Syndrome. IQR: interquartile range, 25% to 75%

### Rabies test results by municipality and animal species

The immunofluorescence and MIT tests, their positivity, and the calculated Ratio of Positives, grouped by host species, and their totals, are presented in Table 2. Of the 260 tests performed, 156 were negative in the first stage; 144 of these were submitted to the MIT. Cattle, when compared to horses, besides being the most tested species, were responsible for most positive results (93.3% of all positives), and they had the highest Ratio of Positives (Table 2). In contrast, the odds ratio of detecting bovine positives by MIT were 1%, whereas the odds ratio of detecting equine positives by MIT were 42.9%. The NS test numbers were nearly constant across years and between animal species and ranged between: (a) 40 to 66 by year; (b) 32 to 48 for bovines; (c) seven to 18 for equines.

**Table 2:** Number of rabies diagnosis tests performed in each tecnhique, divided by tested herbivore, in the State of Rio de Janeiro, within the period of 2020 to 2024. The Ratio of Positives is the total positives divided by the number of Immunofluorescence tests done

Herbivore tested	Immunofluorescences		MIT		Total Positives	Ratio of Positives
	*n*	Positives	*n*	Positives	*n*	%
Bovine	204	97	95	1	98	48.0
Equine	56	7	49	3	10	17.9
Total	260	104	144	4	108	41.5

MIT: Mice inoculation technique

The NS cases per municipality are shown in map A of Fig. 2, where it is clear that there is a concentration of the diagnostic requests on the border shared with Minas Gerais State, both in the Northwestern region and in the Médio Paraíba region. These are the same regions with the largest pasture areas (map B, Fig. 2). The NS cases are concentrated in cattle and horses on the border with Minas Gerais is followed by a high Ratio of Positives mainly in northwestern region, although the Médio Paraíba region also presents many municipalities with a high Ratio of Positives (named in map B, Fig. 2).

### Correlations between neurological syndrome cases, rabies tests results, and municipal land use

The number of NS cases showed a positive correlation with municipalities’ areas of pasture (Spearman’s rho = 0.209, *p* = 0.038). The number of rabies positives was also strongly positively correlated with the pasture area (Spearman’s rho = 0.233, *p* = 0.001) and negatively correlated with the shrub area (Spearman’s rho = -0.202, *p* = 0.026). The Ratio of Positives did not show a relationship with any land use.

### Linear relations among neurological syndrome cases, rabies tests results, and municipal land use

**Fig. 1:**
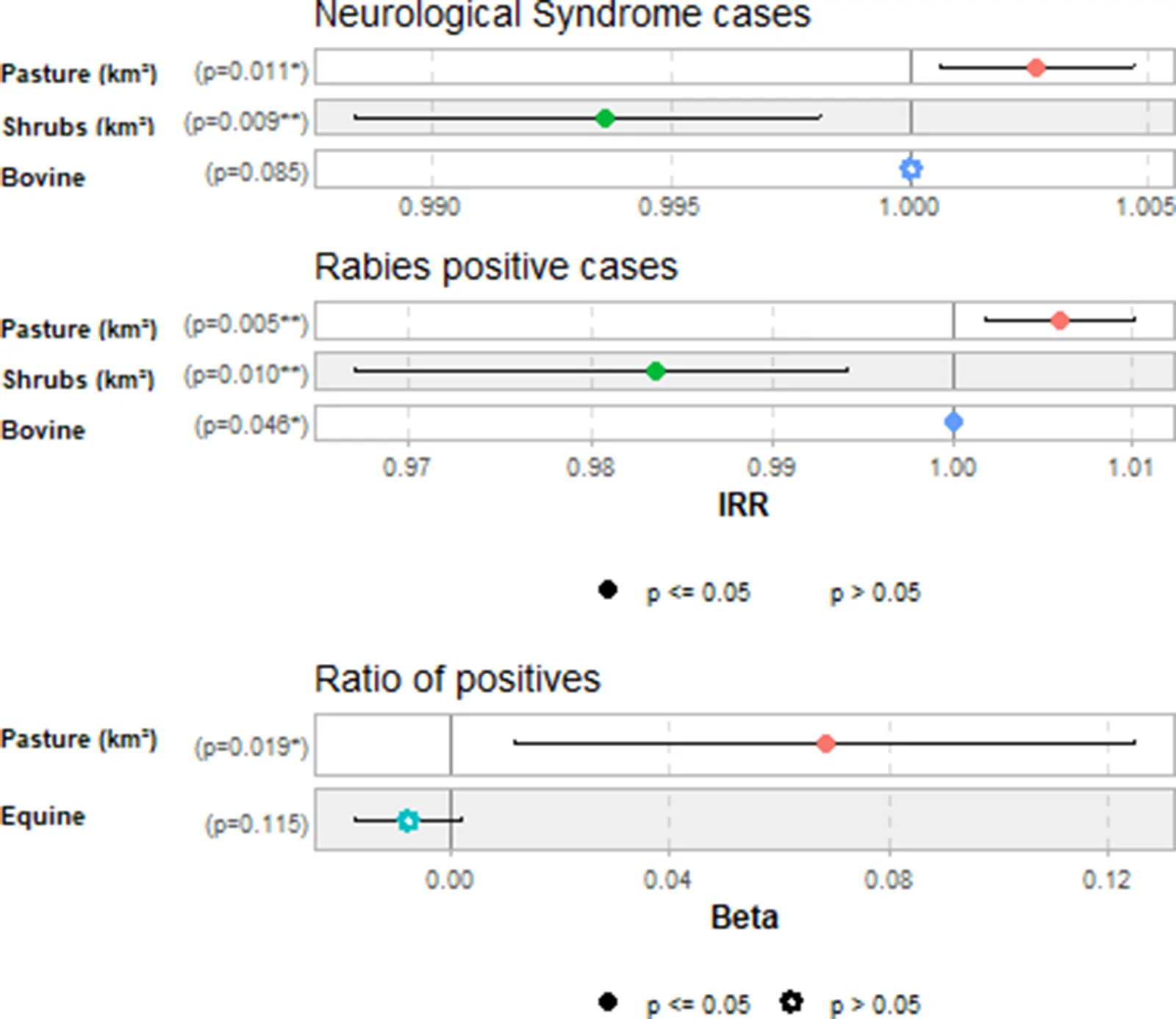
Generalized linear models results over the dependent variables considered. Neurological syndrome cases were fitted over a Poisson distribution, Rabies positive cases were fitted over a Negative Binomial distribution, and Ratio of Positives were fitted over a Gaussian distribution. Graphs show the Incidence Rate Ratios (IRR) for the NS cases and Rabies positives, and Beta values for the Ratio of Positives. Confidence intervales are the horizontal bars and p values are shown for each factor considered at the models

The generalized linear regression model indicated that the number of NS cases was positively associated with municipal pasture area (*p* = 0.011) and negatively associated with shrub area (*p* = 0.009) and cattle population size, which spite of non-signficant increased model fit (*p* = 0.085). The same factors influenced the number of rabies-positive tests: greater pasture area increased the count (*p* = 0.005), whereas larger shrub area (*p* = 0.010) and larger cattle populations (*p* = 0.046) were associated with decreases. The linear model for the ratio of positives showed a significant positive association with municipal pasture area (*p* = 0.019); a larger equine population was weakly associated with a decrease in the ratio (*p* = 0.115), and although this effect was not statistically significant it improved model fit. Incidence rate ratios (IRRs), regression coefficients (β) and 95% confidence intervals from the generalized linear models predicting NS cases, number of positives, and ratio of positives are shown in Fig. 1.

The average value and standard deviation of the pixel area in the State of Rio de Janeiro were 829 +/- 3m2, and final areas were converted to square kilometers. The median ratio between cattle and horse populations per municipality (34233 and 1299 animals, respectively, the total medians sum 35532 animals) and the pasture area (208.49 km^2^) was 170 animals per square kilometer; the median for Shrub area was 0.7154 km^2^. Based on these values the municipalities considered for this study have a base risk of presenting 1.68 cases of NS and 0.56 rabies cases per year. This amount increases proportionately with the municipality’s pasture area, however they decrease with a larger Shrub area and cattle population. Hypothetically, in a municipality with 0.72 km² of shrubs and two bovines grazing over 140 km^2^ of pasture, the risk would increase to 2.18 NS cases per year, with at least one rabies case expected.

## Discussion

This geo-epidemiological analysis of NS case-diagnosis data from municipalities in Rio de Janeiro for 2020–2024 indicates an almost constant risk of NS of non-rabies origin. An additional risk of rabies occurrence – primarily associated with hematophagous bats – was also identified. This additional risk is statistically positively associated with the municipality’s pasture area, negatively associated with its shrub area, and smaller as cattle population enlarges.

This differential risk between bovines and equines may reflect sampling bias, given the marked asymmetry between the cattle herd (> 2.6 million heads in the State during this period) and the much smaller equine population (only 4% of cattle numbers). The equines’ exposure would be diluted within the cattle herd, leading to a smaller number of cases and the Ratio of Positives in horses. Nevertheless, other causes might contribute to the differential risk. The first would be a higher number of NS cases from other causes than the rabies virus in horses than in cattle, decreasing the Ratio of Positives for rabies in those animals. This possibility weakens as it faces the general better care provided to horses compared with a bovine, in production systems (stabled versus pasture).

Another factor would be the existence of different susceptibilities to the rabies infection between cattle and horses, as bovines are more frequently successfully infected facing the same viral challenge. Immunological (age, smaller anti-rabies vaccination proportion in cattle), as well as environmental aspects (pasture cattle’s wider exposure to hematophagous bats when compared to stabled horses), or even behavior-related ones (horses would be more likely to perceive the bat’s attack, reducing both the exposure time and infecting dose) can contribute for this difference. Taking into account the statistical difference of age between cattle and equine populations presenting with NS, older horses would have better immune response than the younger bovines. As a reinforcement of this possibility, it is noticeable that even though horses were less sampled and presented fewer positive cases in general, they have a far higher odds to be diagnosed through MIT (Table 2), which suggests they had lower viral loads. There probably is a causal web among these factors, resulting in the differences of the rabies infection profile between the two species.

**Fig. 2:**
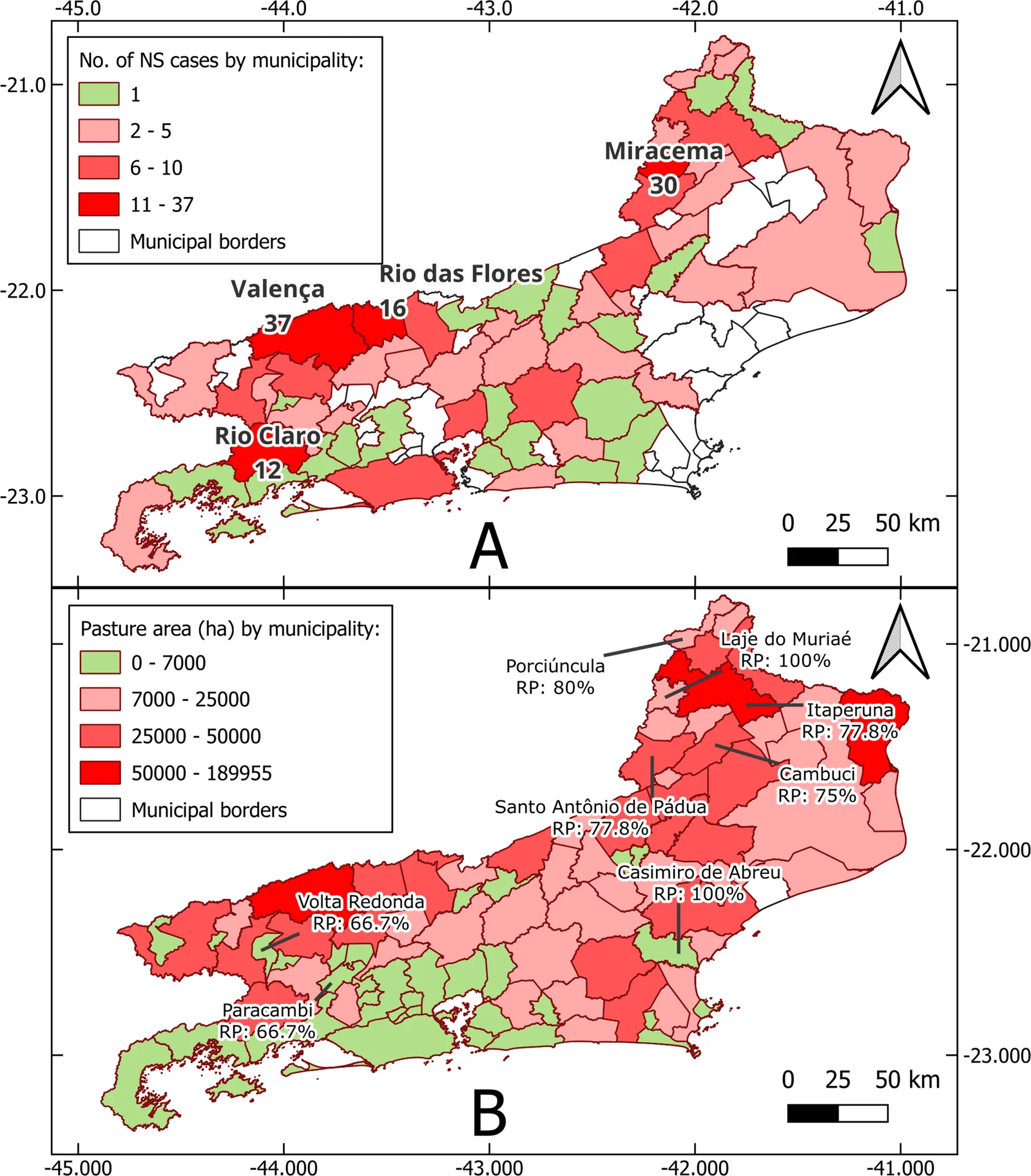
Geospatial distribution among Rio de Janeiro municipalities of: **A**) bovine and equine Neurological Syndrome cases. Municipalities with over 10 tests within the period are named in the map; **B**) Pasture area (ha). Municipalities with Ratio of Positives (RP) of 66.7% or above are named in the map. Year range: 2020–2024. Source: Prepared by Monteiro, RV (2026)

The municipalities’ proportion with at least one animal rabies case (66.3%) is very similar to the 63% reported by Martins et al. (2013). However, as shown in maps A and B of Fig. 2, there are some municipalities with no diagnosis or positive tests surrounded by municipalities with tests and positive results. In these “silent areas”, it is very likely that there is an error in the notification from the farmers and/or insufficient assistance provided by the official veterinary service. Such silent areas were already recognized ten years ago by Martins et al. (2015). This lack of information for some municipalities may bias our results; however, we expect the bias to be small because most municipalities were sampled (Table 1) and they account for the majority of the state’s area (Fig. 2). The geospatial distribution of the Ratio of Positives shows that some municipalities have a way higher value compared to the average of positives (Table 2), some over 75% (Fig. 2). These figures are a result of their great area of Pasture, which may indicate a higher prevalence of rabies virus in those hematophagous bat populations, resulting in greater transmissibility of the virus to the attacked hosts, as well as suggesting greater reach and surveillance by the SVO (Veterinary Health Service), which results in a greater number of collections and referrals for examination of biological material.

Although our research did not include direct measurements of vertebrate diversity in each municipality, the correlations found here support the initial hypothesis that pasture areas limit hematophagous bats’ feeding opportunities because of their low animal diversity, which is inherent to such environments (Fig. 1; Filazzola et al. 2020). The hematophagous bats would have to frequently feed on cattle and horses living in those areas, as these animals are one of the few feeding sources available there. This results in a higher number of NS cases and rabies-positive cases. One agreeing data points to this hypothesis is the actual negative effect of shrub areas over the number of positive results, for such areas are usually abandoned pasture areas or recovery forests, containing a wider animal diversity and without cattle (Fig. 1).

The linear model makes ecological sense: pasture environments reduce animal species diversity, which drives the increase in NS and rabies-positive cases (Fig. 1). The cattle presence increases the risk for both indicators, as they seem to be more susceptible to the rabies infection, but a bigger cattle density tends to dilute the rabies transmission effect to farm animals, as only one bovine is capable of sustaining an entire hematophagous bat colony. Therefore, with a larger number of bovines and equines, there would be only a larger distribution among animals from the same number of hematophagous bats’ attacks.

The change in the type of land use is pointed out to be a cause for emerging diseases since the beginning of this century (Rulli et al. 2025). Examples of land use changes associated with prevalence and incidence increase of infectious diseases in animals have been constantly brought to attention within the scientific literature (Miranda et al. 2022; Perfecto et al. 2023; Rulli et al. 2025). The data analysis presented here supports the idea that emerging diseases can result from changes in land use. For rabies specifically, this is a preliminary framework that requires testing in other years and states before any practical application. Once validated, it could provide a quantitative, predictive measure of risk to help establish criteria for more targeted epidemiological surveillance and rabies control.

## Data Availability

The datasets generated during and/or analysed during the current study are not publicly available due to government restrictions but are available from the corresponding author on reasonable request.
